# Prioritizing cases from a multi-institutional cohort for a dataset of pathologist annotations

**DOI:** 10.1016/j.jpi.2024.100411

**Published:** 2024-11-16

**Authors:** Victor Garcia, Emma Gardecki, Stephanie Jou, Xiaoxian Li, Kenneth R. Shroyer, Joel Saltz, Balazs Acs, Katherine Elfer, Jochen Lennerz, Roberto Salgado, Brandon D. Gallas

**Affiliations:** a U.S. Food and Drug Administration, Center for Devices and Radiological Health, Office of Science and Engineering Laboratories, Division of Imaging, Diagnostics, and Software Reliability, Silver Spring, MD, United States of America; b Department of Pathology and Laboratory Medicine, Emory University, Atlanta, GA, United States of America; c Department of Pathology, Renaissance School of Medicine, Stony Brook University, Stony Brook, NY, United States of America; d Department of Oncology and Pathology, Cancer Centre Karolinska (CCK), Karolinska Institutet, Stockholm, Sweden; e Department of Clinical Pathology and Cancer Diagnostics, Karolinska University Hospital, Stockholm, Sweden; f Division of Cancer Prevention, National Cancer Institute, National Institute of Health, Shady Grove, MD, United States of America; g BostonGene, Waltham, MA, USA; h Division of Research, Peter Mac Callum Cancer Centre, Melbourne, Australia; i Department of Pathology, ZAS Hospitals, Antwerp, Belgium

**Keywords:** Data, Sampling, Prioritization, Validation

## Abstract

**Objective:**

With the increasing energy surrounding the development of artificial intelligence and machine learning (AI/ML) models, the use of the same external validation dataset by various developers allows for a direct comparison of model performance. Through our High Throughput Truthing project, we are creating a validation dataset for AI/ML models trained in the assessment of stromal tumor-infiltrating lymphocytes (sTILs) in triple negative breast cancer (TNBC).

**Materials and methods:**

We obtained clinical metadata for hematoxylin and eosin-stained glass slides and corresponding scanned whole slide images (WSIs) of TNBC core biopsies from two US academic medical centers. We selected regions of interest (ROIs) from the WSIs to target regions with various tissue morphologies and sTILs densities. Given the selected ROIs, we implemented a hierarchical rank-sort method for case prioritization.

**Results:**

We received 122 glass slides and clinical metadata on 105 unique patients with TNBC. All received cases were female, and the mean age was 63.44 years. 60% of all cases were White patients, and 38.1% were Black or African American. After case prioritization, the skewness of the sTILs density distribution improved from 0.60 to 0.46 with a corresponding increase in the entropy of the sTILs density bins from 1.20 to 1.24. We retained cases with less prevalent metadata elements.

**Conclusion:**

This method allows us to prioritize underrepresented subgroups based on important clinical factors. In this manuscript, we discuss how we sourced the clinical metadata, selected ROIs, and developed our approach to prioritizing cases for inclusion in our pivotal study.

## Introduction

In this work, we demonstrate a level of detail for slide sourcing and case sampling that is beyond what is found in the literature but is recommended in a regulatory submission.^1^ Such details are important for device reviewers to understand the data-collection methods and dataset characteristics to confirm data independence and representativeness of the patient population. We want to demonstrate how we addressed underrepresented clinical and demographic subpopulations with the details of our multi-institutional slide-sourcing and case sampling methods. We expect this information will be interesting to pathologists that are not currently involved in research, as the odds are increasing that they will be recruited to efforts to create data for artificial intelligence/machine learning (AI/ML) model training and testing.

Our High Throughput Truthing (HTT) project, is an interdisciplinary and multi-stakeholder collaboration of clinicians and scientists with research interests in AI/ML and breast cancer (BC).^2^, ^3^, ^4^, ^5^, ^6^, ^7^ The HTT project's goal is to develop a validation dataset for computational models for the assessment of stromal tumor-infiltrating lymphocytes (sTILs) in triple negative breast cancer (TNBC). With our intention to submit this dataset for qualification through the FDA's Medical Device Development Tools (MDDT) Program,^8^ we believe transparency in the data collection process is important and not often described in detail in the literature. As a multi-institutional dataset, our dataset will allow direct performance comparisons of AI/ML models and assess their generalizability as an external validation dataset.^9^, ^10^, ^11^, ^12^

In our pilot study, we observed a predominant distribution of TNBC cases with low sTILs densities.^2^, ^3^, ^4^ We were concerned that our final dataset would be dominated by cases with low sTILs densities and other prevalent subgroups and may not give a full performance picture of a model's generalizability to the entire clinical population. To address this concern, we developed a hierarchical rank-sort method to prioritize a diverse dataset that includes underrepresented subgroups of important clinical factors, such as patient demographics, sTILs densities, and disease attributes. In this manuscript, we describe our efforts to source clinical metadata, to select regions of interest (ROIs) from whole slide images (WSIs), and to prioritize cases for inclusion in our pivotal study.

TNBC is a BC that lacks the expression of estrogen receptors (ERs), progesterone receptors (PRs), and human epidermal growth factor receptor 2 (HER2), which precludes the targeted hormonal therapy options of other BC subtypes.^13^^,^^14^ TNBC has a worse prognosis among BC subtypes^15^ as well as a high risk of local recurrence.^16^ More prevalent seen in younger females.^17^ TNBC has higher than average rates of disease for patients younger than 55 years old^18^^,^^19^ with the reported age at diagnosis including averages between 47.52^20^ and 62.6^21^ years. TNBC is commonly described to be more prevalent among Black or African American individuals, who have a nearly 2-fold increase in relative rates of TNBC as compared to White individuals.^17^^,^^22^^,^^23^ Fortunately, sTILs have been demonstrated to be prognostic and predictive biomarkers in TNBC,^24^, ^25^, ^26^, ^27^, ^28^, ^29^, ^30^, ^31^, ^32^, ^33^, ^34^ with an increased sTILs density correlating with longer patient survival.^30^, ^31^, ^32^, ^33^, ^34^

Given the increasing prognostic evidence for sTILs and that AI/ML developers are developing algorithms to perform the sTILs assessment,^18^^,^^35^, ^36^, ^37^, ^38^, ^39^ we chose this biomarker as our use case for dataset creation. Using the International Immuno-Oncology Biomarker Working Group on Breast Cancer's proposed standardized approach for the sTILs assessment,^27^^,^^40^ we designed a dataset for the validation of ROI-based approaches to the sTILs density estimation^2^, ^3^, ^4^ from hematoxylin and eosin (H&E)-stained core biopsies of TNBC with corresponding scanned WSIs and clinical metadata. We present our approach to prioritizing cases for inclusion in our pivotal study and creating an inclusive dataset enriched for underrepresented subpopulations.

## Material and methods

### Inclusion criteria

All selected cases were H&E-stained sections of TNBC core biopsies stained within the last 7 years. We focused our case collection on core biopsies to ensure a dataset of pre-therapeutic tissue samples. For each case, we obtained the H&E-stained glass slide, a scanned WSI from each home institution's available digital WSI scanner(s), and corresponding clinical metadata.

### Clinical metadata

Our project collaborators selected the clinical metadata elements using consensus and supporting literature after receiving feedback from the FDA's Medical Device Development Tools (MDDT) Program during our initial interactions with the Agency.^5^ The goal of the collected metadata is to provide a description of the study population. Some requested clinical elements, such as patient age, sex, race, ethnicity, BC stage, and WSI scanner make and model, are intended to characterize the patients in the study cohort. Other elements allow us to better understand our dataset and allow for secondary analyses. Some of the features extend beyond the standard of care and include various pathological features (histological subtypes, Nottingham grade, mitosis (/10HPF), nuclear grade, tubule formation, BRCA mutation, Ki-67 percentage, Ki-67 intensity, Ki-67 stain manufacturer, PD-L1 Combined Composite Score, PD-L1 Combined Composite Score Test), additional clinical attributes (tumor size (cm), TNM (tumor, node, metastasis) stage), and WSI features (image resolution, numerical aperture, and objective magnification) when available. The table of metadata elements with their descriptors and expected values, our project's data dictionary, can be found in Appendix B.

### Data extraction

As an interdisciplinary, collaborative project, the HTT project includes scientists and clinicians from government, academia, and industry from both within and outside the USA. Two of our collaborators volunteered to source slides for our pivotal study, Emory University Hospital and Stony Brook University Hospital. Emory University Hospital is a 733 bed, quaternary care, academic medical center^41^^,^^42^ in DeKalb County, GA, which has an estimated population of 763,000. DeKalb county is comprised of 54.6% Black or African American, 36% White, 6.5% Asian, and 0.1% Native Hawaiian and Other Pacific Islander individuals with 14.5% of the population in poverty.^43^ Stony Brook University Hospital is a 624 bed, tertiary care, academic medical center^44^ in Suffolk County, NY, which has an estimated population of 1.5 million. Suffolk County is comprised of 83.6% White, 9% Black or African American, 4.9% Asian, 0.1% Native Hawaiian and Other Pacific Islander individuals with 6.4% of the population in poverty.^45^ Emory cases were obtained from two care sites within the hospital network, whereas the SB cases were from a single site.

**Fig. 1:**
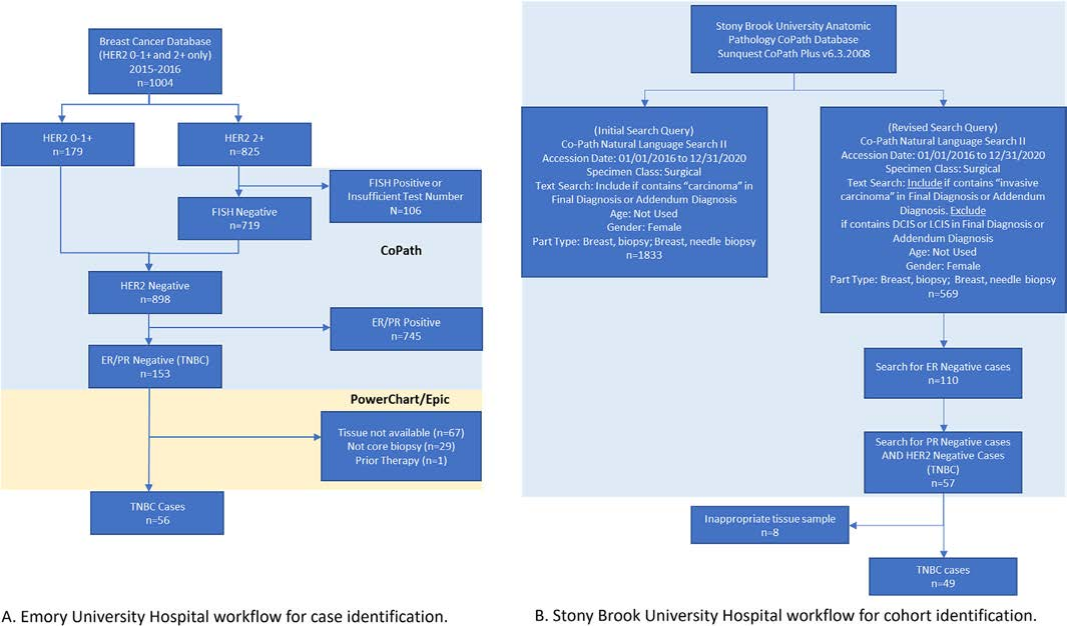
Institutional workflows for cohort identification. A. (Emory University Hospital) Patients identified through manual chart review beginning with an institutional breast cancer database. Provided database only included cases of HER2 0–1+ (negative) and HER2 2+ (equivocal). B. (Stony Brook University Hospital) Patients identified from Anatomic Pathology Database using natural language queries. The blue background denotes steps performed using the institution's pathology information system: CoPath used in both institutions. The yellow background denotes steps involving the electronic health record: Powerchart and Epic used at Emory. “*n*” refers to the count of unique patients. (For interpretation of the references to colour in this figure legend, the reader is referred to the web version of this article.)

With the provided inclusion criteria and data dictionary (Appendix B), each site used their own local protocols for case identification, which were dependent on institutional resources and workflows. The Emory data extraction team consisted of one research fellow under the supervision of a clinical lead. The SB data extraction team involved two clinical leads, two research team members, and a Honest Broker. Both teams had assistance from their corresponding pathology lab staff for slide identification, preparation, and digitization. All H&E slides were deidentified, scanned by the institution's WSI scanner, and shipped to the FDA team. WSIs and clinical metadata were shared after deidentification in accordance with HIPAA Safe Harbor standards.^46^ Fig. 1 shows a comparison of the institutional workflows.

#### Emory University Hospital

At Emory, the research fellow was provided with a list of all patients between 2015 and 2016 with a primary diagnosis of BC and HER2 immunohistochemistry staining results of 0–1+ (HER2 negative) or 2+ (HER2 equivocal). For the HER2 equivocal cases, we confirmed their final HER2 status using results from fluorescence in situ hybridization analysis; only the final reports were reviewed. A specimen is considered HER2 gene amplified if either the HER2 copies number per cell is ≥6 or if both the HER2 copies number per cell is ≥4 and the HER2 fluorescent signals to chromosome 17 centromere signals ratio is ≥2.^47^ The ER and PR statuses were extracted using the pathology reports. HER2 status, ER, PR status, and some metadata information (sex, Ki-67 intensity and percent-positive, diagnosis, and Nottingham grade) were determined using the laboratory information system CoPath (Oracle Cerner, Kansas City, MO). The electronic health record (EHR), PowerChart (Oracle Cerner, Kansas City, MO) and Epic (Verona, WI), was used to obtain the remaining clinical metadata elements and confirm that the sample met study criteria. Two EHRs were used due to an institutional data management system change. The Emory research fellow reviewed all relevant tissues blocks and excluded cases that did not have an available core biopsy of the primary breast tumor that could be used to prepare a new H&E slide. A pathologist reviewed the final tissue blocks before the new H&E slides were cut. All data were de-identified before data transfer to the FDA team.

#### Stony Brook University Hospital

At SB, the research team identified TNBC cases using a natural language query of pathology reports from 2015 to 2020 using Sunquest CoPath Plus (software version 6.3.2008, Clinisys, Inc., Tuscon, AZ). See the Supplementary Materials for the full query. Tissue blocks of the identified cases were reviewed by one of the clinical leads to determine the final set of cases to be provided to the FDA team. In addition, a team member queried SB's Cancer Registry for available clinical metadata for the selected patients. The registry consisted of clinical data provided by SB to the North American Association of Central Cancer Registries (NAACCR).^48^ To request these elements from the registry, we mapped our metadata to the NAACCR's data dictionary (version 18); these mapped elements are found in the Supplementary Materials. Another research team member then reviewed the retrieved NAACCR data and the pathology information system's (CoPath) pathology reports to identify any remaining metadata. Data were reviewed by an Honest Broker to confirm proper deidentification before data transfer.

### FDA-cleared WSI scanner

Given that we intend to submit our dataset for qualification under the MDDT program, our WSIs must be produced using FDA-cleared scanners to be used in regulatory submissions, which our collaborators did not have. Therefore, we engaged the digital pathology services team in the Department of Pathology at Ohio State University's (OSU) Wexner Medical Center and paid them to scan our pivotal study slides on an FDA-cleared Aperio AT2 DX scanner. We chose the Aperio AT2 Dx scanner becasue it uses a WSI format (.svs) that is supported by non-proprietary image viewers. After the FDA team received the glass slides, we performed a nominal quality check of the glass slides, and then shipped the slides to OSU for scanning. OSU performed basic quality checks of the glass slides and scanned images before returning the materials. We then performed a nominal quality check of all the scanned images. All glass slides were scanned on an Aperio AT2-DX WSI scanner at 40× equivalent magnification with a numeric aperture of 0.95 mm (0.25 μm per pixel).

### Clinical metadata processing

Clinical metadata processing included harmonizing each institution's data to the data dictionary's expected values. Data harmonization included removing abbreviations, standardizing data types to the expected format, and handling additional data such as comments and diagnoses. To capture disease severity, we mapped the TNM stage to aggregated anatomic BC stages of I, II, III, and IV. We capture the variance of sTILs density bins using entropy, which increases as categorical labels are more evenly distributed.^49^ Data processing and descriptive statistics were generated using R (version 4.0.0) and RStudio (version 1.3.1056) (Posit Software, PBC, Boston, MA).

### Region of interest selection

We used a panel of expert readers (XL, BA, RS) to select ROIs from the obtained WSIs. Experts were defined as collaborators familiar with the task who helped create the project's training materials.^4^ We divided the 86 WSIs among 7 expert readers with a selection goal of 10 ROIs per WSI. Each reader provided a first-pass estimate of the sTILs density for the selected ROI. These sTILs densities are used in the hierarchical rank-sort method as preliminary estimates for the actual sTILs density that will be determined by additional annotators. The protocol for ROI selection is provided in the Supplementary Materials and includes capturing the following tissue features: tumor region type, percentage of tumor-associated stroma (0%–100%), sTILs density (0%–100%), and, where applicable, identifiable pitfalls in the sTILs assessment.^4^ For ROI selection, the experts used an implementation of caMicroscope, an open-source online digital WSI viewer and annotation platform,^50^ hosted by Emory University.

### Case prioritization - batch creation

Once ROI selection was completed, we created a hierarchical rank-sort method^51^ to prioritize cases, and then we organized the cases into batches of eight cases (WSIs) for our pivotal study. Organizing the cases into batches allows us to track and distribute study participant assignments that can be completed in a reasonable amount of time; a batch of eight cases with 10 ROIs per WSI takes approximately 30–60 min. The hierarchical rank-sort method also allows us to prioritize, or enrich, underrepresented subgroups. Considering the clinical features and annotations of our initial sample prevalence, we created the hierarchical rank-sort method as outlined in Table 1.

**Table 1:** Our hierarchical rank-sort approach to batch creation. Each column represents a variable in the sort method, and its value is assigned a point between 0 and 2. Once all values are computed, WSIs are sorted by rank (highest to lowest) from left to right in the table in a hierarchical, sequential, way. “BCS” is an abbreviation of “Breast Cancer Score.”

Points	Normalized Density Count Score	Race and Ethnicity Score	BCS, Age, Sex Score	Pitfall Score
2	$\frac{\text{High density count ROIs}}{\text{Total}\#\text{ROIs in WSIs}}$	Race ≠ White Ethnicity = Hispanic	BCS = III, IV Age ≤ 40 41 ≤ Age ≤ 50 81 ≤ Age ≤ 90 Sex ≠ F	Rare pitfalls (≤2 cases) - Carcinoma In Situ - Ischemia - Sparse Distribution - Fibers - Over Staining - Under Staining

1	$\frac{\text{Medium density count ROIs}}{\text{Total}\#\text{ROIs in WSIs}}$		BCS = I, II 51 ≤ Age ≤ 60 71 ≤ Age ≤ 80	Semi-rare pitfalls (3–10 cases) - Benign Glands - Necrosis/Fibrin - Eosinophilia - Perinuclear Clearing - Crush Artifact

0	$\frac{\mathrm{Low}\;\text{density count ROIs}}{\text{Total}\#\text{ROIs in WSIs}}$	Race = White Ethnicity = Not Hispanic	BCS = NA 61 ≤ Age ≤ 70 Sex = F	Common pitfalls (>10 cases) - Adipocytes - Nerves/Vessels - Pyknotic Nuclei - Fibroblasts

We sorted the WSI cases by the scores assigned to variable values defined in each column of Table 1. “Normalized Density Count Score” represents the fraction of ROIs in the WSI within an sTILs density bin (low: x ≤ 10%; medium: 10% < x ≤ 40%; high: 40% < x). Each density bin fraction is multiplied by its assigned point value (prioritizing High density ROIs) and summed together to create a WSI-level score. For columns “Race and Ethnicity Score” and “BCS, Age, and Sex Score,” we assign a point for each variable present for a WSI (prioritizing underrepresented groups) and sum the points within each category. As an example of this type of scoring, a WSI of a “White” and “Non-Hispanic” patient receives 0 points for the “Race and Ethnicity Score,” whereas a WSI of a “Black or African American” and “Hispanic” patient would receive four points. This logic is similarly applied to the “BCS, Age, and Sex Score.” Last, the “Pitfall Score” variable is a WSI-level score of the aggregate count of pitfalls present in the ROIs for that WSI. The total amount of each pitfall is multiplied by the assigned point value (prioritizing rare pitfalls) and added together to create the “Pitfall Score.”

After all variables have been calculated, we sorted the cases in the following order: Normalized Density Count Score > Race and Ethnicity Score > BCS, Age, and Sex Score > Pitfall Score. The hierarchical component of the rank is invoked when there is a tie in a score variable. Given that some WSIs were from the same patient, we kept the WSI with the higher overall rank and excluded the lower ranked WSI(s). This means that we have only one WSI per patient in our pivotal study. The top 40 cases (WSIs) were selected for the first 5 batches for our pivotal study using the group assignment of 1, 2, 3, 4, 5, 1, 2, 3, etc.

## Results

### Glass slides and whole slide images

To date, we have received a total of 122 glass slides that meet our inclusion criteria. SB provided 66 slides from 49 unique patients identified between the years 2015 and 2020. We were given multiple slides for a subset of patients with sequential tissue slices of the same biopsy. Emory provided 56 slides of unique patients from 2015 to 2016. Both institutions provided newly cut and stained slides. We are currently developing and validating the statistical analysis plan for validating an AI model with this data. The results will inform the final size of the dataset.

### Clinical metadata

For the 122 glass slides, we received clinical metadata on a total of 105 unique patients with TNBC. Due to practice patterns, the full clinical TNM stage, and thus ultimately BC stage, was not always available from the core biopsy report. We report on the missingness of received data on the five clinical elements that were considered when performing data sampling in Table 1: age, sex, race, ethnicity, and BC stage.

All received cases were female, and the mean age was 63.44 years. 60% of all cases were White patients, 38.1% were Black or African American, and 1.9% were unknown. While examining the racial distribution by site, SB cases were 87.8% White with 10.2% Black or African American, and Emory cases were 35.7% White with 62.5% Black or African American. For both sites, Not Hispanic or Latino was the ethnic majority with a combined prevalence of 91.4% of the provided cases.

**Fig. 2:**
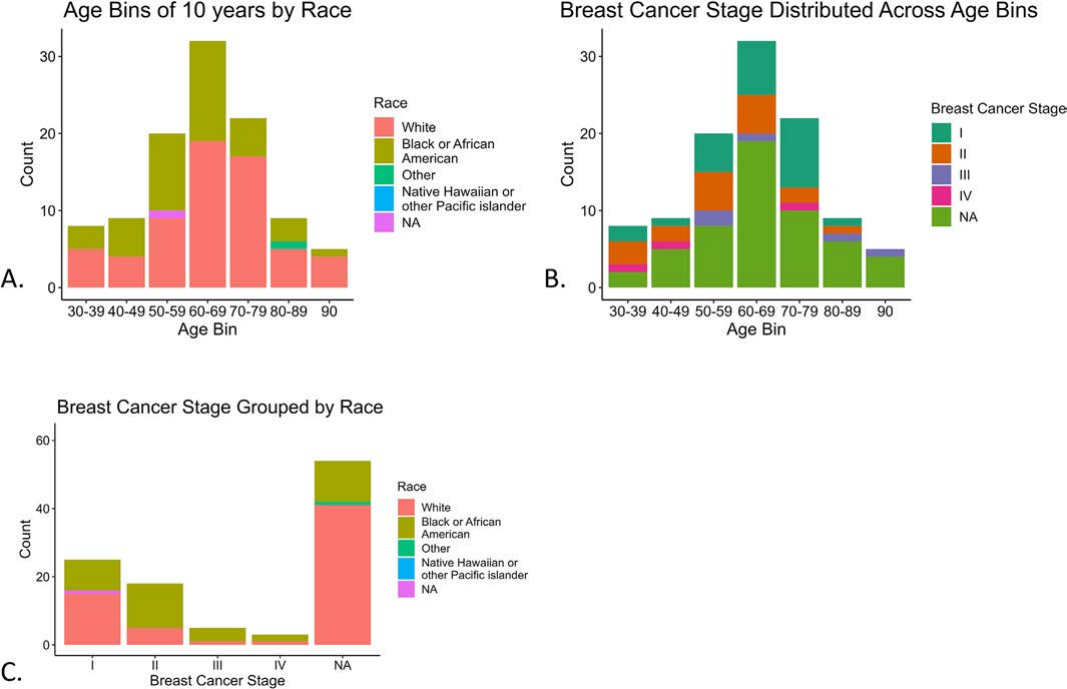
Clinical metadata distributions. A. Age distribution (decade binned) by Race. B. Distribution of age (decade binned) by Breast Cancer Stage. C. Distribution of breast cancer stages by Race.

Table 3 shows the demographic breakdown of cases by site. Fig. 2A shows the age distribution by race. See Table 1, Table 2, Table 3 and Fig. 1 of the Supplementary Materials for additional descriptive statistics on other collected variables.

**Table 2:** Table of percent missingness of select clinical metadata at the patient level. Any unknown values are treated as a missing value. SB stands for Stony Brook University Hospital.

Feature	All sites (*n* = 105)	SB (*n* = 49)	Emory (*n* = 56)
Age	0% (*n* = 0)	0% (*n* = 0)	0% (*n* = 0)
Sex	0% (*n* = 0)	0% (*n* = 0)	0% (*n* = 0)
Race	1% (*n* = 1)	0% (*n* = 0)	1.8% (*n* = 1)
Ethnicity	3.8% (*n* = 4)	0% (*n* = 0)	7.1% (*n* = 4)
Breast cancer stage	51.4% (*n* = 54)	83.7% (*n* = 41)	23.2% (*n* = 13)

**Table 3:** Demographic distribution of cases with breakdown by site. Age is represented as the mean value with the standard deviation. Sex, race, and ethnicity are total counts with percentages. Any unknown values are treated as a missing (NA) value. SB stands for Stony Brook University Hospital.

		All Sites (*n* = 105)	SB (*n* = 49)	Emory (*n* = 56)
Age (mean (SD))		63.44 (14.35)	64.98 (14.95)	62.09 (13.80)
Sex (%)	Female	105 (100.0)	49 (100.0)	56 (100.0)
Race (%)	White	63 (60.0)	43 (87.8)	20 (35.7)
	Black or African American	40 (38.1)	5 (10.2)	35 (62.5)
	Other	1 (1.0)	1 (2.0)	0 (0.0)
	NA	1 (1.0)	0 (0.0)	1 (1.8)
Ethnicity (%)	Not Hispanic or Latino	96 (91.4)	45 (91.8)	51 (91.1)
	Hispanic or Latino	5 (4.8)	4 (8.2)	1 (1.8)
	NA	4 (3.8)	0 (0.0)	4 (7.1)

Table 4 shows the distribution of BC stages by site, and Fig. 2B shows the distribution of BC stage by age. Both sites have a higher prevalence of lower BC stage and cases with unknown BC stage. Of the known tumor sizes, the mean size is 2.40 cm (SD 1.64), and the median size is 2.00 cm (IQR 1.30–3.10), where SD and IQR are standard deviation and interquartile range, respectively. See Fig. 2 in the Supplementary Materials for a histogram of tumor size stratified by BC stage. Fig. 2C shows the distribution of race by BC stage, which highlights that we have cases of various BC stages for both White and Black or African American populations.

**Table 4:** Table of distribution of Breast Cancer Stage by site.

Breast Cancer Stage	Overall	SB	EUH
n (%)	(*n* = 105)	(*n* = 49)	(*n* = 56)
I	25 (23.8)	4 (8.2)	21 (37.5)
II	19 (18.1)	3 (6.1)	16 (28.6)
III	5 (4.8)	1 (2.0)	4 (7.1)
IV	3 (2.9)	1 (2.0)	2 (3.6)
NA	53 (50.5)	40 (81.6)	13 (23.2)

### Batch creation from selected ROIs

At the time of ROI selection, we had collected 86 WSIs while simultaneously procuring additional cases. SB had provided 67 WSIs for their 49 unique patients. Emory had provided 19 WSIs for 19 unique patients. We distributed the 86 WSIs among our experts for ROI selection, and ROI selection and annotation were completed on 50 cases. The remaining will be saved for future annotation. From the 50 WSIs, 501 ROIs were annotated. 397 ROIs were Evaluable for the sTILs assessment with 269 low sTILs density ROIs, 88 medium sTILs density ROIs, and 40 high sTILs density ROIs. The remaining 104 ROIs were Not Evaluable. The Evaluable and Not Evaluable determination is the first step in the sTILs assessment and captures whether the tissue type within the ROI is appropriate for the sTILs assessment (is there tumor nearby). Only Evaluable cases will have an sTILs density.

**Fig. 3:**
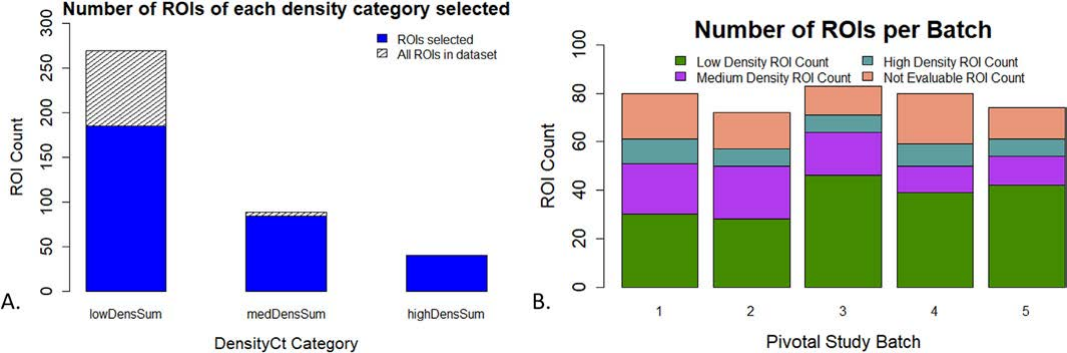
Results of hierarchical rank-sort method. A) Number of ROIs within each sTILs density bin for all 55 WSIs. Blue indicates ROIs included in the pivotal study batches by the hierarchical rank-sort method. Hashed regions represent all ROIs within that sTILs density bin including those not included in the pivotal study batches. B) Distribution of sTILs densities bins across pivotal study batches after sorting. (For interpretation of the references to colour in this figure legend, the reader is referred to the web version of this article.)

**Fig. 4:**
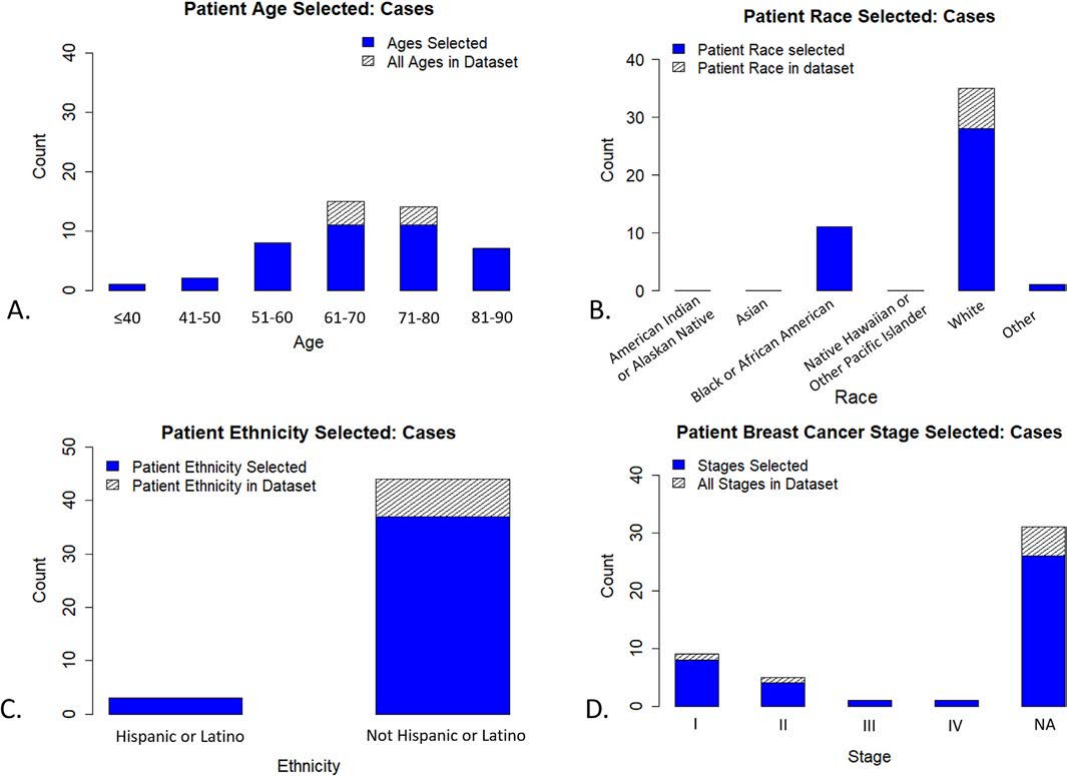
Characteristics of selected cases (WSIs). This figure shows the unique distribution of the patient demographic features (age, race, and ethnicity) and disease stages included in the pivotal study batches by the hierarchical rank-sort method. Our approach retained the patients within the lowest frequency age bins and disease stage, all “Black or African American” patients (13/47), and all “Hispanic or Latino” patients (5/47).

**Fig. 5:**
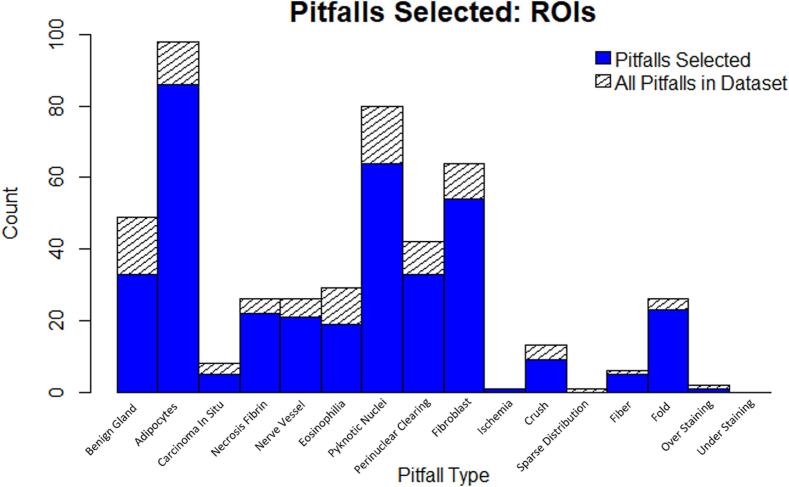
Distribution of Pitfalls in the full set of ROIs selected and annotated and in the pivotal study batches selected by the hierarchical rank-sort method.

In the figures below, we show distributions of the Evaluable ROIs and cases selected in the first five batches and in the full dataset. Before performing ROI selection, the sTILs density distribution had a skewness of 0.60 with an entropy of 1.20 for distribution of sTILs density bins. After ROI selection, the skewness improved to 0.46 and the entropy increased to 1.24. See Figs. 3–6 in the Supplemental Materials for a plot of the cumulative distribution and histograms of the sTILs densities before and after ROI Selection. Fig. 3A shows the number of selected ROIs for each sTILs density bin, and Fig. 3B demonstrates the distribution of sTILs densities across study batches. Fig. 4A–D show the distributions of age, race, ethnicity, and BC stage. Fig. 5 shows the distribution of identified pitfalls in the sTILs assessment. The figures demonstrate that our hierarchical rank-sort method creates batches that preferentially sample cases with high sTILs density ROIs and patients that are demographically underrepresented in our dataset.

## Discussion

This manuscript presents our methods for sourcing and prioritizing cases for a dataset of pathologist sTILs density annotations in TNBC from WSI images of H&E glass slides. We present the pipeline we used to collect 122 slides of 105 unique patients from two different academic medical institutions and the hierarchical rank-sort method to prioritize cases into pivotal study batches. Considering our data's purpose as a validation dataset, we are most interested in a diverse dataset that prioritizes underrepresented subgroups of important clinical factors. This prioritization allows for better insight into model performance across a range of subpopulations because the true distribution of clinical factors may not allow for this level of analysis.

Each site developed their own data collection protocol given the inclusion and exclusion criteria, which could have contributed to variability in the collected data. For example, SB used a natural language query, which was a streamlined and responsive approach that required fewer personnel hours. Emory's manual collection approach identified more cases but with a much slower collection time.

Regardless of the impact of the institutions' data collection approach, having patients from geographically diverse institutions yielded a broader distribution than if sourced from a single institution. Our sampled population has a 2:1 ratio of White:non-White patients. Though this ratio does not match published summary statistics of TNBC, we are currently still receiving data from Emory. Our dataset's mean age of 63.44 years (median age: 64 years) has 29.5% of patients with an age less than or equal to 55 years. Though our dataset may be considered an older population relative to some reported incidence ages of TNBC,^17^, ^18^, ^19^, ^20^, ^21^^,^^52^ it is close to the 70–74 age group, which is the peak incidence rate age group as reported in the US SEER database.^53^ Though the age and racial distributions of our dataset does not feature explicit concordance with the known published literature, it's distribution similarities of age and race between our dataset and the US population gives us confidence that our data does not omit key subgroups.

During ROI selection, we used spreadsheets alongside the viewer for the first pass. This led to missing and mismatched data, causing the uneven distribution of ROIs per batch in Fig. 3B. We have resolved this problem by integrating ROI selection within the viewer and adding checkpoints for data collection. This limitation challenged us to focus efforts on further optimizing our cases with whatever data were available. In addition, with the prioritization of underrepresented groups, we address the natural overrepresentation of low sTILs densities and other clinical metadata elements, as demonstrated from our own pilot study experience^3^^,^^4^ and documented literature.^32^^,^^54^, ^55^, ^56^, ^57^, ^58^, ^59^ Had we a large enough dataset, we would be able to perform a statistically powered subgroup analysis, but given our constraints on data availability, a not uncommon challenge, our batch creation approach is our best solution. Prioritizing underrepresented groups provides a robust validation dataset for understanding the performance in future subgroup analyses.

Our pivotal study is not without its limitations. As we noted earlier, we have limited and missing data, either from a systemic cause like institutional data collection methods or from more random causes like volunteer underperformance. The applied hierarchical rank-sort method prioritized cases with complete metadata, and missing data was treated with a weight of 0. This reflects the challenges of a retrospective review to identify cases of TNBC. Given that we did not check for any discrepancies between core biopsies samples and resection specimens, there may be false positive cases in the dataset; however, we do not believe this substantially impacts the methodology of performing the sTILs assessment. Another limitation is that our dataset is currently comprised of only two hospital networks, and the lack of a site variable may introduce a selection bias. As we continue with additional slide sourcing, we will look for new clinical sites to support acquiring a representative dataset.

## Conclusions

The High Throughput Truthing project's goal is to create an external dataset for the validation of a model's performance on the sTILs assessment. To achieve this, we are prioritizing cases from underrepresented subgroups to allow insight into the model's performance in a variety of subpopulations, not just those most frequently encountered. We further prioritize the inclusion of medium and high sTILs densities and less frequent pitfalls and clinical attributes. This enrichment promotes a robust assessment of AI models by providing high quality ground truth validation data from diverse sites and diverse populations that are still representative of key features in TNBC.

## Disclaimer

The mention of commercial products, their sources, or their use in connection with material reported herein is not to be construed as either an actual or implied endorsement of such products by the Department of Health and Human Services. This is a contribution of the U.S. Food and Drug Administration and is not subject to copyright.

## Disclosures

The authors report no conflicts related to the current work. The funders had no role in the design of the study; in the collection, analyses, or interpretation of data; in the writing of the manuscript; or in the decision to publish the results. XL has served as an advisor for Astra Zeneca, Roche, Eli Lilly and Onviv, and Champions Oncology has funded research in his lab. JS acknowledges support from NCI award 5UH3CA225021and is co-owner of Chilean Wool. RS receives non-financial support from Merck, Case 45, and Bristol Myers Squibb (BMS); research support from Merck, BMS, Puma Biotechnology and Roche; and personal fees from Roche, BMS, Astra Zeneca, Daicchii Sankyo and Exact Sciences for advisory boards. KS serves as a member of the scientific Advisory Board with financial interests for KDx Diagnostics.

## Declaration of competing interest

The authors declare the following financial interests/personal relationships which may be considered as potential competing interests:

Emma Gardecki reports financial support was provided by Oak Ridge Institute for Science and Education. Stephanie Jou reports financial support was provided by FDA's Office of Women's Health. Brandon Gallas reports financial support was provided by FDA Office of Women's Health. Balazs Acs reports financial support was provided by The Swedish Society for Medical Research (Svenska Sällskapet för Medicinsk Forskning). Xiaoxian Li reports a relationship with Astra Zeneca that includes:. Xiaoxian Li reports a relationship with Roche that includes:. Xiaoxian Li reports a relationship with Eli Lilly that includes:. Xiaoxian Li reports a relationship with Onviv that includes:. Xiaoxian Li reports a relationship with Champions Oncology that includes: funding grants. Joel Saltz reports a relationship with National Cancer Institute that includes: funding grants. Joel Saltz reports a relationship with Chilean Wool that includes:. Roberto Salgado reports a relationship with Merck that includes:. Roberto Salgado reports a relationship with Case 45 that includes:. Roberto Salgado reports a relationship with Bristol Myers Squibb that includes: consulting or advisory. Roberto Salgado reports a relationship with Puma Biotechnology that includes:. Roberto Salgado reports a relationship with Roche that includes: consulting or advisory. Roberto Salgado reports a relationship with Astra Zeneca that includes: consulting or advisory. Roberto Salgado reports a relationship with Daicchii Sankyo that includes: consulting or advisory. Roberto Salgado reports a relationship with Exact Sciences that includes: consulting or advisory. Kenneth Shroyer reports a relationship with KDx Diagnostics that includes: The following co-authors are members of the National Editorial Board of the Journal of Pathology Informatics: Joel Saltz and Jochen Lennerz. If there are other authors, they declare that they have no known competing financial interests or personal relationships that could have appeared to influence the work reported in this article.

## Figures and Tables

**Table B1:** Required metadata.

Feature	Description	Expected values
Age	Age of patient at time of sample acquisition. If patient >89 years of age, Age reported as 90.	##
Sex	Patient's sex.	Female Male
Race	Patient's race. More than one response allowed.	American Indian or Alaska Native Asian Black or African American Native Hawaiian or Other Pacific Islander White
Ethnicity	Patient's ethnicity	Hispanic or Latino Not Hispanic or Latino
Breast Cancer Stage	Denotes Breast Cancer Stage at time of biopsy	0 I II III IV
WSI image resolution	How much slide area is captured in 1 pixel, described as micrometers/pixel or microns per pixel (mpp).	##
WSI scanner make	Manufacturer name of the whole slide imaging scanner.	(Character string)
WSI scanner model	Model of whole slide imaging scanner used.	(Character string)
WSI numerical aperture	Aperture diameter of microscope lens used during image acquisition. Reported in millimeters (mm)	##
WSI objective magnification	Magnification of microscope objective lens used during image acquisition. Reported as a multiplier (##X)	##
Specimen collection site	Site where the tissue was collected	(Character string)
Slide preparation site	Site where slides were prepared	(Character string)
Slide scanning site	Site where slides were scanned	(Character string)

We have mapped the metadata fields to the NAACCR (North American Association of Central Cancer Registries) separately and can be found in the Supplemental Materials.

**Table B2:** Optional metadata.

Feature	Description	Expected values
Tumor size (cm)	Size of tumor in image as measured in centimeters - CT or Mammogram before surgery around time of biopsy	##
TNM stage	Denotes clinical tumor staging at time of biopsy	T(0–4)N(0–3)M(0–1)
Histological subtypes	The histological classification of breast cancer type.	Ductal, NOS Lobular Tubular Cribriform Mucinous Micropapillary Apocrine Metaplastic Adenoid cystic Other
Nottingham grade	Histological grade determined by degree of tubule formation, nuclear pleomorphism, and mitotic activity.	I (well-differentiated) II (moderately differentiated) III (poorly differentiated)
Mitosis (/10HPF)	Count of number of mitosis in 10 high powered fields (40× magnification) or per unit area (2 mm^2^) in the most active part of the tumor.	1 (0–6 mitosis/2 mm^2^) 2 (7–12 mitosis/2 mm^2^) 3 (13 mitosis or more/2 mm^2^)
Nuclear grade	Degree of nuclear pleomorphism in tissue sampled including nucleus size, shape, and uniformity.	1 (small, regular uniform cells) 2 (moderate increase in size and variability) 3 (marked variation)
Tubule formation	Proportion of tumor displaying tubular structure.	1 (Majority of tumor, >75%) 2 (Moderate degree, 10–75%) 3 (Little or none, <10%)
BRCA mutation	Patient with either positive BRCA1 or BRCA2 mutation as detected by germline testing. Optional.	BRCA1 BRCA2 Negative N/A (Test not performed)
Ki-67 percentage	Percentage of cells that are Ki-67 positive. Optional.	## N/A (Stain not performed)
Ki-67 intensity	Intensity of Immunohistochemistry (IHC) stain. Optional.	1+ (Weak positive staining) 2+ (Moderate positive staining) 3+ (Strong positive staining) N/A (Stain not performed)
Ki-67 stain manufacturer	Manufacturer of Ki-67 stain.	(Character string)
PD-L1 combined composite score	(Number of PD-L1 positive cells (tumor cells, lymphocytes, macrophages) / total number of tumor cells) * 100	##
PD-L1 combined composite score test	What companion diagnostic test was used to determine the PD-L1 Combined Composite Score.	(Character string)

The optional metadata is a “wish list”, and we recognize that each institution has different processes and data to share.

## Data Availability

The HTT project pilot study data is available on this public repository: https://github.com/DIDSR/HTT. The data underlying this article is not publicly available as we continue to collect data.

## Appendix B Inclusion criteria and data dictionary

**Inclusion criteria**

• Core biopsies of TNBC (ER/PR/HER2 negative)
• Slides that have been stained with hematoxylin and eosin within the last 7 years

**Exclusion criteria**

• Tissue collected after administration of any therapy (e.g., neoadjuvant, chemotherapy, radiation therapy).

**Note:** The below is a refined and reformatted version of the data requested from participating sites. Some fields were added based on slide sourcing from multiple sites and additional fields collected during slide scanning. We also marked additional WSI image details for inclusion into the planned MDDT proposal.

## Appendix A Supplementary data

The supplementay materials contain additional descriptive statistics, Stony Brook University's natural language query, ]NACCR mappings, and the ROI selection protocol. Supplementary material
