# Supplementary material for: Prioritizing cases from a multi-institutional cohort for a dataset of pathologist annotations
Source: J Pathol Inform. 2024 Nov 16;16:100411. doi: 10.1016/j.jpi.2024.100411 (PMC11667696; doi:10.1016/j.jpi.2024.100411)
Supplement: Supplementary file 1 — The supplementay materials contain additional descriptive statistics, Stony Brook University's natural language query, ]NACCR mappings, and the ROI selection protocol. [file mmc1.docx]

# Supplemental Materials

## Table 1. Frequency of Histologic Subtypes Among Received Cases.

Any unknown values are treated as a missing value which is represented in the table as “N/A”. We present the distributions with and without “N/A”s.

| **Histologic Subtype** | **Count** | **Percent with N/A** | **Percent without N/A** |
| --- | --- | --- | --- |
| Apocrine | 6 | 12.77% | 20.69% |
| Ductal, NOS | 18 | 38.30% | 62.07% |
| Favor Ductal, NOS | 1 | 2.13% | 3.45% |
| Favor Metaplastic | 1 | 2.13% | 3.45% |
| Metaplastic | 1 | 2.13% | 3.45% |
| Suspicious Metaplastic | 1 | 2.13% | 3.45% |
| Unknown | 1 | 2.13% | 3.45% |
| N/A | 18 | 38.30% | -- |
| **Total Count** | **47** | **47** | **29** |

## Table 2. Frequency of Nottingham Grades Among Received Cases

Any unknown values are treated as a missing value which is represented in the table as “N/A”. We present the distributions with and without “N/A”s.

| **Nottingham Grade** | **Count** | **Percent with N/A** | **Percent without N/A** |
| --- | --- | --- | --- |
| 1 | 3 | 6.38% | 6.98% |
| 2 | 7 | 14.89% | 16.28% |
| 2-3 | 3 | 6.38% | 6.98% |
| 3 | 30 | 63.83% | 69.77% |
| N/A | 4 | 8.51% | -- |
| **Total Count** | **47** | **47** | **43** |

## Table 3. Frequency of BRCA Mutation Among Received Cases

Any unknown values are treated as a missing value which is represented in the table as “N/A”. We present the distributions with and without “N/A”s.

| **BRCA Mutation** | **Count** | **Percent with N/A** | **Percent without N/A** |
| --- | --- | --- | --- |
| Positive | 0 | 0% | 0% |
| Negative | 2 | 4.56% | 100% |
| N/A | 45 | 95.74% | -- |
| **Total Count** | **47** | **47** | **2** |

## Figure 1. Histogram of Ki67 Percentages Among Received Cases


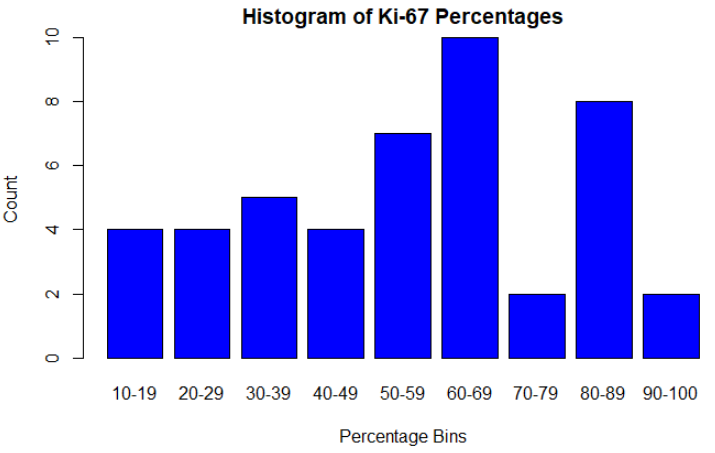


Figure 1 is a histogram of Ki67 percentages among received cases. The percentages were grouped in bins of size 10. The Ki-67 Intensity of received cases was 3+ for 46 cases and unknown (N/A) for 1 case.

## Figure 2: Distribution of Breast Cancer Stages by Tumor Size (cm).


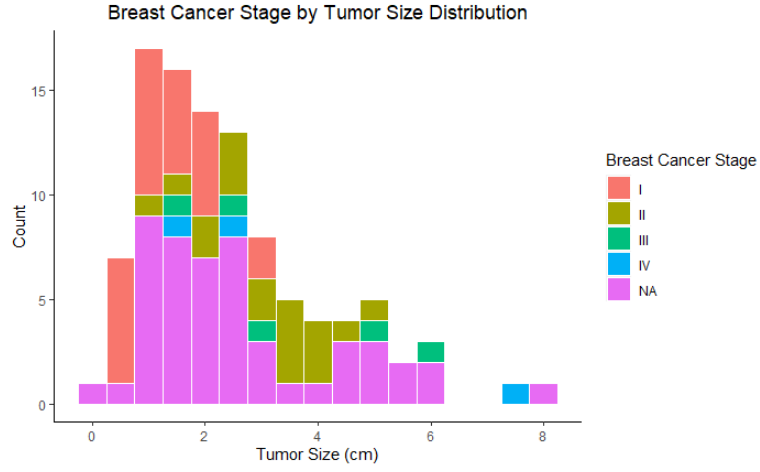


Figure 2 is a histogram of tumor sizes stratified by breast cancer stage. Cases where the breast cancer stage was unknown are represented by “N/A”. Four cases had unknown tumor size.

### Figure 3: Plot of the Cumulative Distributions of sTILs Densities Pre- and Post-ROI Selection


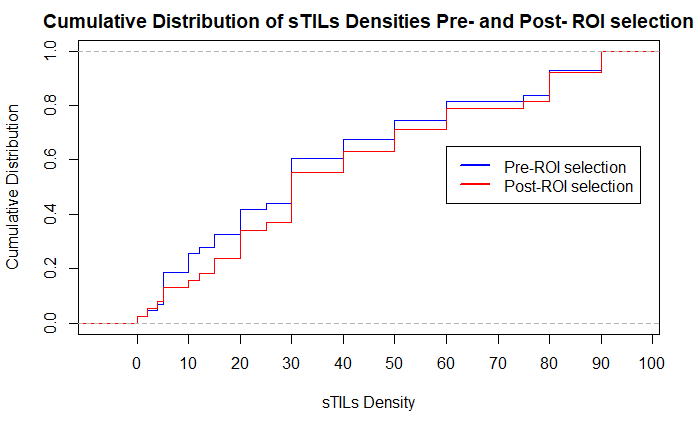


Figure 3 is a plot of the cumulative distributions of stromal tumor-infiltrating lymphocyte (sTILs) densities pre- (blue line) and post- (red line) ROI selection.

### Figure 4: Histogram of sTILs Density in dataset before ROI Selection


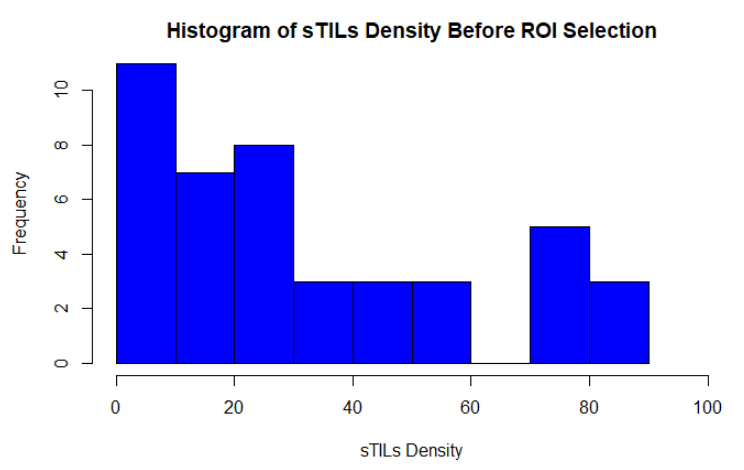


Figure 4 is a histogram of stromal tumor-infiltrating lymphocyte (sTILs) densities before ROI selection. sTILs densities were grouped into bins of size 10.

### Figure 6: Histogram of sTILs Density in dataset after ROI Selection


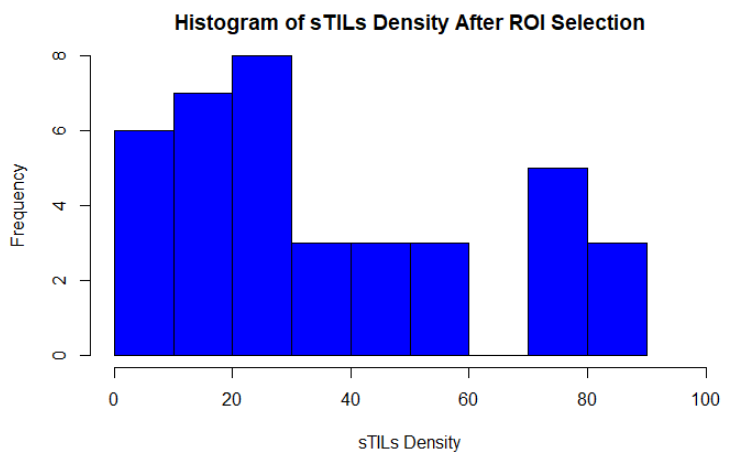


Figure 5 is a histogram of stromal tumor-infiltrating lymphocyte (sTILs) densities after ROI selection. sTILs densities were grouped into bins of size 10.

# Natural Language Processing Query

**Search Strategy for Triple Negative Breast Cancer Biopsies**

1. Searched the Stony Brook University Hospital Anatomic Pathology clinical case database using Sunquest CoPath Plus software version 6.3.2008
2. Utilized CoPath Natural Language Search II (no race) search feature
3. Initial Search Query:
   1. Accession Date: 01/01/2016 to 12/31/2020
   2. Specimen Class: Surgical
   3. Text Search: Include if contains “carcinoma” in Final Diagnosis or Addendum Diagnosis
   4. Age: Not Used
   5. Gender: Female
   6. Part Type: Breast, biopsy; Breast, needle biopsy
4. Initial results: 1833 cases
5. Revised Search Query:
   1. Changed Text Search to include if contains ”invasive carcinoma” in Final Diagnosis or Addendum Diagnosis
   2. Changed Text Search to exclude if contains DCIS or LCIS in Final Diagnosis or Addendum Diagnosis
   3. Results: 569 cases (on 2621 pages)
   4. Searched these results for ER:Negative cases: total of 110
   5. Searched 110 ER:Negative cases for those also diagnosed as PgR:Negative AND Her2 Negative

*Final Results: 57 triple negative breast core biopsy cases from 2016-20*

# NACCR Mapping

#sex

{1:'Male', 2:'Female', 3:'Other (intersex, disorders of sexual development/DSD)', 4:'Transsexual, NOS', 5:'Transsexual, natal male', 6:'Transsexual, natal female', 9:'Not stated/Unknown'}

#Race1

{01:'White', 02:'Black', 03:'American Indian, Aleutian, or Alaska Native (includes all indigenous populations of the Western hemisphere)', 04:'Chinese', 05:'Japanese', 06:'Filipino', 07:'Hawaiian', 08:'Korean', 10:'Vietnamese', 11:'Laotian', 12:'Hmong', 13:'Kampuchean (Cambodian)', 14:'Thai', 15:'Asian Indian or Pakistani, NOS (code 09 prior to Version 12)', 16:'Asian Indian', 17:'Pakistani', 20:'Micronesian, NOS', 21:'Chamorro/Chamoru', 22:'Guamanian, NOS', 25:'Polynesian, NOS', 26:'Tahitian', 27:'Samoan', 28:'Tongan', 30:'Melanesian, NOS', 31:'Fiji Islander', 32:'New Guinean', 96:'Other Asian, including Asian, NOS and Oriental, NOS', 97:'Pacific Islander, NOS', 98:'Other', 99:'Unknown'}

#Race2

{01:'White', 02:'Black', 03:'American Indian, Aleutian, or Alaska Native (includes all indigenous populations of the Western hemisphere)', 04:'Chinese', 05:'Japanese', 06:'Filipino', 07:'Hawaiian', 08:'Korean', 10:'Vietnamese', 11:'Laotian', 12:'Hmong', 13:'Kampuchean (Cambodian)', 14:'Thai', 15:'Asian Indian or Pakistani, NOS (code 09 prior to Version 12)', 16:'Asian Indian', 17:'Pakistani', 20:'Micronesian, NOS', 21:'Chamorro/Chamoru', 22:'Guamanian, NOS', 25:'Polynesian, NOS', 26:'Tahitian', 27:'Samoan', 28:'Tongan', 30:'Melanesian, NOS', 31:'Fiji Islander', 32:'New Guinean', 88:'No further race documented', 96:'Other Asian, including Asian, NOS and Oriental, NOS', 97:'Pacific Islander, NOS', 98:'Other', 99:'Unknown', NULL:'Race 2-5 not coded'}

#Race3

{01:'White', 02:'Black', 03:'American Indian, Aleutian, or Alaska Native (includes all indigenous populations of the Western hemisphere)', 04:'Chinese', 05:'Japanese', 06:'Filipino', 07:'Hawaiian', 08:'Korean', 10:'Vietnamese', 11:'Laotian', 12:'Hmong', 13:'Kampuchean (Cambodian)', 14:'Thai', 15:'Asian Indian or Pakistani, NOS (code 09 prior to Version 12)', 16:'Asian Indian', 17:'Pakistani', 20:'Micronesian, NOS', 21:'Chamorro/Chamoru', 22:'Guamanian, NOS', 25:'Polynesian, NOS', 26:'Tahitian', 27:'Samoan', 28:'Tongan', 30:'Melanesian, NOS', 31:'Fiji Islander', 32:'New Guinean', 88:'No further race documented', 96:'Other Asian, including Asian, NOS and Oriental, NOS', 97:'Pacific Islander, NOS', 98:'Other', 99:'Unknown', NULL:'Race 2-5 not coded'}

#Race4

{01:'White', 02:'Black', 03:'American Indian, Aleutian, or Alaska Native (includes all indigenous populations of the Western hemisphere).', 04:'Chinese', 05:'Japanese', 06:'Filipino', 07:'Hawaiian', 08:'Korean', 10:'Vietnamese', 11:'Laotian', 12:'Hmong', 13:'Kampuchean (Cambodian)', 14:'Thai', 15:'Asian Indian or Pakistani, NOS (code 09 prior to Version 12)', 16:'Asian Indian', 17:'Pakistani', 20:'Micronesian, NOS', 21:'Chamorro/Chamoru', 22:'Guamanian, NOS', 25:'Polynesian, NOS', 26:'Tahitian', 27:'Samoan', 28:'Tongan', 30:'Melanesian, NOS', 31:'Fiji Islander', 32:'New Guinean', 88:'No further race documented', 96:'Other Asian, including Asian, NOS and Oriental, NOS', 97:'Pacific Islander, NOS', 98:'Other', 99:'Unknown', NULL:'Race 2-5 not coded'}

#Race5

{01:'White', 02:'Black', 03:'American Indian, Aleutian, or Alaska Native (includes all indigenous populations of the Western hemisphere)', 04:'Chinese', 05:'Japanese', 06:'Filipino', 07:'Hawaiian', 08:'Korean', 10:'Vietnamese', 11:'Laotian', 12:'Hmong', 13:'Kampuchean (Cambodian)', 14:'Thai', 15:'Asian Indian or Pakistani, NOS (code 09 prior to Version 12)', 16:'Asian Indian', 17:'Pakistani', 20:'Micronesian, NOS', 21:'Chamorro/Chamoru', 22:'Guamanian, NOS', 25:'Polynesian, NOS', 26:'Tahitian', 27:'Samoan', 28:'Tongan', 30:'Melanesian, NOS', 31:'Fiji Islander', 32:'New Guinean', 88:'No further race documented', 96:'Other Asian, including Asian, NOS and Oriental, NOS', 97:'Pacific Islander, NOS', 98:'Other', 99:'Unknown', NULL:'Race 2-5 not coded'}

# raceCodingSysCurrent

{1:'4-value coding: 1 = White, 2 = Black, 3 = Other, 9 = Unknown', 2:'SEER < 1988 (1-digit)', 3:'1988-1990 SEER & CoC (2-digit)', 4:'1991-1993 SEER & CoC (added codes 20-97, additional Asian and Pacific Islander codes)', 5:'1994-1999 SEER & CoC (added code 14, Thai)', 6:'2000+ SEER & CoC (added code 88 for Race 2, 3, 4, and 5)', 7:'2010+ SEER & CoC (added codes 15, 16, and 17; removed 09)', 9:'Other'}

# raceCodingSysOriginal

{9:'Other', 1:'4-value coding: 1 = White, 2 = Black, 3 = Other, 9 = Unknown', 2:'SEER < 1988 (1-digit)', 3:'1988-1990 SEER & CoC (2-digit)', 4:'1991-1993 SEER & CoC (added codes 20-97, additional Asian and Pacific Islander codes)', 5:'1994-1999 SEER & CoC (added code 14, Thai)', 6:'2000+ SEER & CoC (added code 88 for Race 2, 3, 4, and 5)', 7:'2010+ SEER & CoC (added codes 15, 16, and 17; removed 09)'}

# raceNapiia

{01:'White', 02:'Black', 03:'American Indian, Aleutian, or Eskimo (includes all indigenous populations of the Western Hemisphere)', 04:'Chinese', 05:'Japanese', 06:'Filipino', 07:'Hawaiian', 08:'Korean', 10:'Vietnamese', 11:'Laotian', 12:'Hmong', 13:'Kampuchean (Cambodian)', 14:'Thai', 15:'Asian Indian or Pakistani, NOS (code 09 prior to Version 12)', 16:'Asian Indian', 17:'Pakistani', 20:'Micronesian, NOS', 21:'Chamorro/Chamoru', 22:'Guamanian, NOS', 25:'Polynesian, NOS', 26:'Tahitian', 27:'Samoan', 28:'Tongan', 30:'Melanesian, NOS', 31:'Fiji Islander', 32:'New Guinean', 96:'Other Asian, including Asian, NOS and Oriental, NOS', 97:'Pacific Islander, NOS', 98:'Other', 99:'Unknown', NULL:'Algorithm was not run'}

#computedEthnicity

{0:'No match was run (for 1994 and later tumors)', 1:'Non-Hispanic last name and non-Hispanic maiden name', 2:'Non-Hispanic last name, did not check maiden name or patient was male', 3:'Non-Hispanic last name, missing maiden name', 4:'Hispanic last name, non-Hispanic maiden name', 5:'Hispanic last name, did not check maiden name or patient was male', 6:'Hispanic last name, missing maiden name', 7:'Hispanic Maiden name (females only) (regardless of last name)', NULL:'1993 and earlier tumors, no match was run'}

#computedEthnicitySource

{0:'No match was run, for 1994 and later tumors', 1:'Census Bureau list of Spanish surnames, NOS', 2:'1980 Census Bureau list of Spanish surnames', 3:'1990 Census Bureau list of Spanish surnames', 4:'GUESS Program', 5:'Combination list including South Florida names', 6:'Combination of Census and other locally generated list', 7:'Combination of Census and GUESS, with or without other lists', 8:'Other type of match', 9:'Unknown type of match', NULL: '1993 and earlier tumors, no match was run'}

#spanishHispanicOrigin

{0:'Non-Spanish; non-Hispanic', 1:'Mexican (includes Chicano)', 2:'Puerto Rican', 3:'Cuban', 4:'South or Central American (except Brazil)', 5:'Other specified Spanish/Hispanic origin (includes European; excludes Dominican Republic)', 6:'Spanish, NOS; Hispanic, NOS; Latino, NOS', 7:'Spanish surname', 8:'Dominican Republic', 9:'Unknown whether Spanish or not'}

# ­­caMicroscope Process Guide and ROI Selection Protocol

## Goal of ROI selection

The overarching goal of ROI selection is to perform a first pass assessment of an image to identify and annotate 10 ROIs for multiple future readers to evaluate. The first-pass ROIs and annotations of each image are summarized per WSI and used to prioritize images for the pivotal study. We prioritize images with the least common demographic and pathologic metadata. More information on the batch selection method is available by request.

## Platform Instructions

1. From the Collection List, select an ROI selection collection by clicking on the blue button labeled “ROI Selection”.
2. Once in the collection, **click “Select ROIs” to begin selecting ROIs for sTILs evaluation**. The number of ROIs already existing on a slide may vary and your “ROIs” column will update as you complete the task. If you want to review the ROIs already selected for an image, click “Review”.
3. To move around the WSI: Use your machine’s **pan and zoom** actions or adjust the magnification and location using the image navigator controls in the bottom right.
4. To select an ROI: **Double-click** a location **to create or reposition** an ROI.
   1. The location of your click will be the center of the new ROI.
   2. We recommend **all annotations be made using 20x magnification**.
   3. If you don’t like the location of the ROI, double-click to reset the position. Annotation data will be reset.
5. Click **“Save & Next”** to save the ROI and corresponding annotation. You can then create a new ROI on the WSI or exit the image using the “Quit” button.
   1. **Once an ROI has been saved, it cannot be deleted**. If you don’t like it, create another.

**Note:** To help resolve issues, please grab the url of the page that shows an issue and include it in an [email to the project team](https://didsr.github.io/HTT.home/assets/pages/team) describing the issue. That will help us to find it quickly and investigate. There is also a “Comment” box to record any issues before you save an annotation.

## ROI Selection Protocol

1. Select **10** ROIs per WSI. Any tissue in the WSI is eligible for annotation. However, we aim to create a diverse set of ROIs which contains both anomalies, such as pitfalls and artifacts, as well as high-value sTILs densities. Therefore: **target diverse morphology and sTILs density** (especially high sTILs density) while distributing ROIs across entire tissue. Each ROI can satisfy multiple targets. Not all targets can be satisfied in each WSI. The numbers to select mentioned below are guides.
   In each WSI:
   1. Select 3 ROIs inside tumor with stroma
      *(1 ROI should be at least 25% void of tissue)*
   2. Select 2 ROIs at invasive margin if discernable with stroma
      *(not sure this is possible with biopsies, 1 ROI should be at least 25% void of tissue)*
   3. Select 2 ROIs inside tumor or at margin **without stroma***(not sure there are many or any of these)*
   4. Select 2 ROIs where there is no proximal tumor
      *(normal tissue: outside 500* $\mu$m *tumor margin)*
   5. Select 2 ROIs for each for the 16 pitfalls listed. The pitfalls were identified during sessions to understand pathologist variability and improve pathologist training and instructions for the assessment of sTILs ([Garcia2022_Cancers_v14p2467](https://doi.org/10.3390/cancers14102467)). The pitfalls are discussed and exemplified in the [CME course](https://didsr.github.io/HTT.home/assets/pages/training-2023/cmeCourse), the test with feedback, and the [reference document](https://didsr.github.io/HTT.home/assets/pages/training-2023/feedbackRefDoc).

      **Pitfalls**:

| Benign glandular elements | Cross-sectionally cut fibroblasts |
| --- | --- |
| Adipocytes | Low grade and/or degenerate ischemic tumor cells |
| Carcinoma in situ | Crushed cells |
| Necrosis and fibrin | Sparsely distributed tumor cells |
| Nerves and/or larger caliber blood vessels | Fibers |
| Eosinophilia | Folds |
| Small/pyknotic nuclei | Over-staining |
| Perinuclear clearing | Under-staining |

1. Provide Annotations on selected ROIs. HTT sTILs Study Annotations collected:
2. ROI Type (Evaluable versus Not Evaluable)
3. % Tumor-Associated Stroma
   - 1. **Only for “Evaluable” ROIs.** Input the percent (%) as an integer using either the slide bar or by clicking the % value to the right of the slide bar and typing the value.
4. sTILs Density
   - 1. **Only for “Evaluable” ROIs.** Input the percent (%) as an integer using either the slide bar or by clicking the % value to the right of the slide bar and typing the value.
5. Tissue Type
   - 1. If the ROI Type is “Evaluable”, select either “Tumor with Stroma” or “Invasive Margin”.
     2. If the ROI Type is “Not Evaluable for sTILs”, select either “Inside tumor/ margin with low/ no stroma” or “No Proximal Tumor (within 500 $\mu$m)”.
6. Pitfalls
   - 1. Click the corresponding checkbox of any pitfalls present in the ROI.
7. Additional Comments
   - 1. Any additional comments about the selected ROI and image can be added in this free-text box.

## Overview of caMicroscope Icons


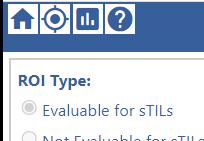


Icon functionality listed in order of left to right:

1. **Home:** Return to list of assigned WSIs and ROIs (Collection List).
2. **ROI Location:** Return to the center of the active ROI. If there is not an active ROI, the browser will alert you with banner message.
3. **TIL Sample:** View the TILs cheat sheet.
4. **ROI Selection Protocol:** View the selection protocol if you need a refresher on anything in this document.
